# Supplementary material for: Depressive symptoms and associated factors among persons with physical disabilities in disability care homes of Kathmandu district, Nepal: A mixed method study
Source: PLOS Glob Public Health. 2023 Jan 12;3(1):e0001461. doi: 10.1371/journal.pgph.0001461 (PMC10021957; doi:10.1371/journal.pgph.0001461)
Supplement: S1 Text — (DOCX) [file pgph.0001461.s001.docx]

**S1 Text. Questionnaire**

Questionnaire in Nepali Language

| lqe'jg ljZjljBfno lrlsT;f zf:q cWoog ;+:yfg dxf/fhuGh lrlsT;f SofDk;  ;fd'bflos lrlsT;f tyf hg:jf:Yo ljefu  k'g:yf{kgf s]Gb\|df /x]sf zfl/l/s ckfËtf ePsf JolQmx?df pbfl;gtfsf] nIf0f / sf/s tTjx? af/] cWoog |
| --- |

| kmf/d g: |  |  |  |
| --- | --- | --- | --- |

| **kl/ro / d~h'/Llgdf .**  gd:t] !  d]/f] gfd k\|lj0f sfsL{ xf] . xfn d lqe'jg ljZjljBfno, lrlsT;f zf:q cWoog ;+:yfg, dxf/fhu~h d]l8sn SofDk;df hg:jf:Yo (BPH) ljifodf :gfts txdf cWoog/t 5' . o;} s\|ddf d o; sf7df08fF} lhNnfdf **k'g:yf{kgf s]Gb\|df /x]sf zfl/l/s ckfËtf ePsf JolQmx?df pbfl;gtfsf] nIf0f / sf/s tTjx? af/] cWoog ub{} 5'** . tkfO{ o; ;DaGwL hfgsf/L lngsf] nfuL 5gf}6df kg'{ePsf] 5 . d tkfO{nfO{ s]lx k\|Zgx? ;f]Wg] 5' . o;sf] nfuL sl/a !) b]vL !% ldg]6 nfUg ;S5 . tkfO{n] lbg' ePsf] hfgsfl/x? o; cWoogsf] nfuL dfq k\|of]u ul/g]5 / uf]Ko /xg]5 . o; cWogdf ;xefuL eP jfkt tkfO{nfO{ k\|ToIf?kn] s'g} xfgL x'g] 5}g / tkfO{sf] ;xefuLttf :jlR5s x'g]5 . tkfO{af6 k\|fKt hfgsfl/x?n] eljiodf o; lhNnfdf s'g} sfo{s\|d ug{ ;xof]u k'¥ofpg] 5 . tkfO{sf] ;xefuLtf o; cWoogsf] nfuL dxTjk'0f{ 5 .  olb tkfO{ nfO{ o; ;DaGwL cGo s'g} lhUof;f ePdf lgw{Ss /fVg ;Sg'x'g]5 .  s] d ca cGt/aftf{ lng ;'? u?+ < |
| --- |
| cGt/aftf{ lbg dGh'/ u/]sf] ==================!-cGt/aftf{ ;'? ug]{ _  cfGtfa{/tf lbg dGh'/ gu/]sf] ===============@ -wGojfb lbO{ cGt/aftf{ glng]_ |

| **s\|=;** | **k\|Zgx?** | **k\|ltlqmof** | **sf]8=g+** | **s}lkmot** |
| --- | --- | --- | --- | --- |
| **s= cfwf/e't hfgsf/L** | | | | |
| != | Gffd÷y/ | ============================================ |  |  |
| @= | pd]/ -k'/f ePsf]_ | =================================================== |  |  |
| #= | lnË | k'?if  dlxnf | !  @ |  |
| **v= ;fdflhs tyf hg;f+lVos hfgsf/L** | | | | |
| $= | hft hflt | blnt  hghftL  dw]zL  d'l:nd  a\|fXd0f If]qL  cGo | !  @  #  $  %  ^ |  |
| %= | wd{ | lxGb'  af}4  lqmlirog  O:nfd  cGo================================ | !  @  #  $  % |  |
| ^= | Zf}lIfs l:ylt | lg/If/  ;fIf/ | !  @ | olb 5}g eg] k\|=g= * |
| &= | olb ;fIf/ ePdf, s'g tx ;Dd k9\g' ePsf] 5 < | cgf}krf/Ls  k\|fylds tx  dfWolds tx  pRr dfWolds tx  :gfts jf ;f] eGbf dfly | !  @  #  $  % |  |
| *= | kl/jf/sf] lsl;d | Psn  ;o'+Qm | !  @ |  |
| (= | j}jflxs l:ylt | lajflxt  clajflxt  ;DaGw laR5]b  ljw'jf / ljw'/ | !  @  #  $ | olb clajflxt  eg] k\|=g= !! |
| !)= | TfkfO{sf] slt cf]6f 5f]/f 5f]/L 5g\ < | ================================================== |  |  |
| !!= | tkfO{{sf] kl/jf/sf] d'Vo cfDbfgLsf] >f]t s] xf] < | s[lif  Jofkf/  Gf]fs/L  Af]blzs ;Dk\|;0f  dhb'/L  cGo eP v'nfpg'xf];\=========== | !  @ # $ % ^ |  |

| !#= | tkfO{sf] cfkmg} k];f 5 sL 5}g < | Nffek\|fKt k];f 5}g  s[lif  >d dhb'/L  Joj;fo  cGo | ! @  #  $  % |  |
| --- | --- | --- | --- | --- |
| !$= | tkfO{sf] 3/kl/jf/af6 cfjZos ;xof]u kfpg'ePsf] 5 < | 5  5}g | ! @ | olb 5}g eg] k\|=g= !^ |
| !%= | olb 5 eg] s:tf] ;xof]u kfpg'ePsf] 5 < | cfly{s  dgf];dflhs  j:t'ut  cGo=============================== | !  @ # $ |  |
| **u :jf:Yo l:ylt** | | | | |
| !^= | xfn tkfO{nfO{ s'g} /f]u ePsf] yfxf 5 < | 5  5}g | !  @ | olb 5}g eg] k\|=g= @) |
| !&= | olb 5 eg] s'g s'g /f]u nfu]sf] 5 < | pRr /Qmrfk  dw'd]x  bd  Uofl:6«s  s'i7/f]u  cGo eP v'nfpg'xf];\============ | ! @ # $ % ^ |  |
| !*= | of] /f]usf] pkrf/ ug'ePsf] 5 < | 5  5}g | ! @ |  |
| !(= | olb 5}g eg], lsg pkrf/ ug'{ gePsf] < | cfly{s l:ylt sdhf]/ eP/  pkrf/ x'G5 eGg] yfxf geP/  dxuf]+ eP/  :jf:Yo ;+:yfdf kx'r geP/  cGo ====================================== | ! @ # $ % |  |
| @)= | s] tkfO{ xfn r'/f]6 lkpg' x''G5 < | 5  5}g | !  @ | olb 5}g eg] k\|=g= @# |
| @!= | olb 5 eg], lbgdf slt j6f r'/f]6 lkpg' x'G5 < | !) j6f eGbf sd  !) b]lv @) j6f  @) j6f eGbf w]/} | !  @ # |  |
| @@= | tkfO{ r/f]6 lsg lkpg'x'G5 < | tgfan] ubf{  nt a;]/ | ! @ |  |
| @#= | tkfO{n] xfn ;'tL{hGo kbfYf{ ;]jg ug'{ x'G5 < | Uf5'{  ulb{g | !  @ |  |
| @$== | ;'tL{hGo kbfYf{ slt slt lbgdf ;]jg ug'{ x'G5 <  -r'/f]6 eGbf Affx]s_ | lbglbg}  slxn] sflx | !  @ |  |
| @%= | s] tkfO{ /S;L lkpg'x'G5 < | 5  5}g | ! @ | olb 5}g eg] k\|=g= @* |
| @^= | olb lkpg'x'G5 eg], slt slt ;dodf lkpg'x'G5 < | ;w}+ h;f]  slxn] sflx |  |  |
| @&= | tkfO{ /S;L lsg lkpg'x'G5 < | tgfa sd x'G5 eg]/  aflg k/]/ | ! @ |  |
| @* | s] tkfO{ of]uf cyjf Wofg ug'{x'G5 < | ;w}+ h;f]  slxn] sflx  ulb{g | !  @  # |  |
| @(= | tkfO{sf] ckfËtf HfGdhft xf] jf hGd kZrft ePsf] xf] < | hGd hft  hGd kZrft | !  @ |  |
| #)= | Zff/L/Ls ckfª\tfsf] k\|sf/ | Kff]lnof]  s'i7/f]u  cªeË ePsf]  hf]lg{ jf d]?b08sf] ;d:o  /Ls]6\;  dfz' ;'Sb} uPsf] -Muscular dystrophy  v'6\6f afªluPsf]  Yffxf 5}g | ! @ # $ %  ^  & * |  |
| #! | s] tkfO{ s'g} k\|sf/sf] ;xfos oGqx?sf] k\|of]u ug'{x'G5 < | Uf5'{  ulb{g | ! @ |  |
| #@ | tkfO{{nfO{ of] k'g:yf{kgf s]Gb\|sf] ef}lts ;ª/rgf slQsf] ;xh dxZf'; ug'{x'G5 < - h:t} lJXnlro/ rnfpg | ;xh 5  ;xh 5}g | !  @ |  |

**a]s l8k|]zg OGe]G6f]/L k|Zs[kof lt nIf0fx? n] tkfO{nfO{ uPsf] @ xKtfdf slQ sf] lk/Nof] /fd|/L ;f]r]/** **hjfkm lbg'xf]nf**

| 1!11  1 | uPsf] b'O{ xKtfdf tkfO{ slQsf] pbf; x'g'x'GYof]< | slxn] eO{g  k\|fo ePF  ;w}+ h;f] ePF  c;x\o ePsf] lyof] | ) ! @ # |  |
| --- | --- | --- | --- | --- |
| @ | uPsf] b'O{ xKtfdf tkfO{ slQsf] lg/fz x'g'eof]< -h:t} cfˆgf] eljiosf] af/]df ;Dem]/_ | slxn] eO{g  k\|fo ePF  ;w}+ h;f] ePF  c;x\o ePsf] lyof] | ) ! @ # |  |
| 3 | uPsf] b'O{ xKtfdf tkfO{nfO{ cfkm\gf] lhjgdf slQsf] c;kmn eP h:tf] nfUof] < | slxn] nfu]g  k\|fo nfUof]  ;w}+ h;f] nfUof]  c;x\o eof]÷k"0f{ ?kn] c;kmn eP h:tf] nfUof] | ) ! @ # |  |
| $ | uPsf] b'O{ xKtfdf tkfO{ cfkm\gf] lhjg b]vL slQsf] c;Gt'i6 x'g' eof]< | slxn] c;Gt'i6 eO{g  k\|fo c;Gt'i6 ePF  ;w}+ h;f] c;Gt'i6 ePF  lhjgsf] x/]s s'/fdf c;Gt'i6 ePF | ) ! @ # |  |
| % | uPsf] b'O{ xKtfdf tkfO{n] cfkm\gf] lhGbuLsf] nflu cfkm'nfO{ slQsf] bf]lif 7fGg' eof]< | slxn] bf]lif 7flgg  k\|fo bf]lif 7fg]+  ;w}+ h;f] bf]lif 7fg]+  lhjgsf] x/]s s'/fdf bf]lif 7fg+] | ) ! @ # |  |
| 6 | uPsf] b'O{ xKtfdf tkfO{n] cfkm\gf] lhGbuLdf slQsf] b'v of ;fl:t ef]Ug' eof]< | slxn] ;fl:t ef]lug  k\|fo ;fl:t ef]u]+  ;w}+ h;f] ;fl:t ef]u]+  lhjgsf] x/]s s'/fdf ;fl:t ef]u]+ | ) ! @ # |  |
| & | uPsf] b'O{ xKtfdf tkfO{nfO{, cfkm'  b]vL cfkm'nfO{ slQsf] jfSs nfUof]< | slxn] jfSs nfu]g  w]/} h;f] jfSs nfUof]  ;w}+ jfSs nfUof]  cfkm'nfO{ g}+ 3[0ff nfUof] | ) ! @ # |  |
| * | uPsf] b'O{ xKtfdf tkfO{ cfkm\gf] ulNtx? k\|lt slQsf] lhDd]jf/ x'g'\eof]< | lhDd]j/ eO{g  ulNt / sdhf]/Lsf] nflu pQ/bfoL ePF  sdhf]/Lx?sf] lgGbf u/]sf] lyPF  x/]s g/fd\|f s'/fk\|lt lhDd]jf/ ePF | ) ! @ # |  |
| ( | uPsf] b'O{ xKtfdf tkfO{n] cfkm'nfO{ slQsf] xfgL k'Øfpg rfxg' eof]< | cfkm'nfO{ s'g} klg xfgL k'Øfpg rfxLg  xfgL k'Øfpg rfx]+ t/ s]xL ug{ ;sLg  d cfkm' nfO{ g} dfg{ rfxGy]+  d}n] df}sf kfpg] lalQs} cfTdxTof uy{]+ | ) ! @ # |  |

| !) | uPsf] b'O{ xKtfdf slQsf ?g'eof] < | Klxn] eGbf a9L /f]Og  klxn] eGbf a9L /f]PF  xKt} e/L /f]PF  rfx]/ klg ?g ;lsg+ | ) ! @ # |  |
| --- | --- | --- | --- | --- |
| !! | uPsf b'O{ xKtfdf tkfO{nfO{ slQsf] emsf]{ nfUof]< | Slxn] eO{g  k\|fo ePF  ;w}+ h;f] ePF  c;x\o ePsf] lyof] | ) ! @ # |  |
| !@ | uPsf] b'O{ xKtfdf tkfO{nfO{ c?;Fu slQsf] xfF; v]n ug{ dg nfUof] < | xfF; v]n ug{ dg nfUof]  klxn] h:tf] xfF; v]n ug{dg nfu]g  af]Ng klg dg nfu]g  s;}sf] s'g} jf:tf /fVg dg nfu]g | ) ! @ # |  |
| !# | uPsf] b'O{ xKtfdf tkfO{n] cfkm\gf] lhjgdf slQsf] lg0f{o lng ;Sg'eof] < | lg0f{o lng ;Sg'eof]  klxn] h:t} lg0f{o lng ;sLg  lg0f{o lng lgs} ufXf] eof]  s'g} klg lg0f{o lng ;sLg | ) ! @ # |  |
| !$ | uPsf] b'O{ xKtfdf tkfO{ nfO{, tkfO{ cfkm' slQsf] /fd\|f] ePF h:tf] nfUof] < | klxn] eGbf g/fd\|f] eO{g  d g/fd\|f] x'Fb} uO/x]sf] h:tf] nfUof]  d ;w}+ g/fd\|f] g}+ eO{/x]+  d s'?k jf l3g nfUbf] b]lvPsf] lyPF | ) ! @ # |  |
| !% | uPsf] b'O{ xKtfdf tkfOn] slQsf] sfd ug{ ;Sg'eof]< | klxn] hlQs} sfd ug{ ;s]+  klxn] h:tf] sfd ug{ ;sLg  sfd ug{ Psbd ufXf] eof]  s]xL klg sfd ug{ ;sLg | ) ! @ # |  |
| !^ | uPsf] b'O{ xKtfdf tkfO{ slQsf] ;'Tg' eof]< | klxn] hlQs} ;'t]+  klxn] h:tf] ;'Tg ;sLg  klxn] eGbf cuf8L lapFlemGy]+ / lgbfpg ;sLg  Psbd} rfF9} lapFlemGy]+ / lgbfOg | ) ! @ # |  |
| !& | uPsf] b'O{ xKtfdf tkfO{ slQsf] yfSg' eof]< | yfSg' ePg  klxnf eGbf a9L yfs]+  h] ubf{ klg yfSby]\+  Psbd} yfs]/ s]lx klg ug{ ;sLg | ) !  @ # |  |
| !* | uPsf] b'O{ xKtfdf tkfO{nfO{ vfgf slQsf] ?rL eof] < | klxn] hlQs} ?rL eof]  klxn] hlt ?rL ePg  Psbd} sd ?rL eof]  k6Ss} ?rL ePg | ) ! @ # |  |
| !( | uPsf] b'O{ xKtfdf tkfO{sf] tf}n slQsf] 36]sf lyof] < | 36]sf] lyPg   - s]hL eGbf a9L 36\of]   !) s]hL eGbf a9L 36\of]  !% s]hL eGbf a9L 36\of] | ) ! @ # |  |
| @) | uPsf] b'O{xKtfdf tkfO{nfO{ cfkm\gf] :jf:Yosf] lrGtf slQsf] nfUof] < | klxnf eGbf a9L lrGtf nfu]g  b'vfO{ / lk8fn] ubf{ lrlGtt lyPF  :jf:Yosf] sf/0fn] ubf{ c? s]lx ;f]Rg ;sLg  :jf:Yosf] sf/0fn] s]xL xf]z g}+ ePg | ) ! @  # |  |
| @! | uPsf] b'O{ xKtfdf tkfO{n] cfkm\gf] lhjg ;fyLsf] af/]df slQsf] ;f]Rg' eof] < | klxnf hlQs} ;f]r]  klxnf hlQs} ;f]rLg  Tolt jf:tf g}+ ePg  jf:tf g}+ ePg | ) ! @ # |  |

**Questionnaire in English**

**BECK DEPRESSION INVENTORY -IA**

**Instructions:** This questionnaire consists of 21 groups of statements. Please read each item carefully, and then pick out the **one statement** in each group that best describes the way you have been feeling during the **past two weeks, including today**. Circle the number beside the statement you have picked. If several statements in one group seem to apply equally well, circle the highest number for that group

**1. Sadness**

- 1. I do not feel sad.
  2. I feel sad much of the time.
  3. I am sad all the time.
  4. I am so sad or unhappy that I can’t stand it.

1. **Pessimism**
   1. I am not discouraged about my future.
   2. I feel more discouraged about my future than I used to be.
   3. I do not expect things to work out for me.
   4. I feel my future is hopeless and was only get worse.
2. **Past Failure**
   1. I do not feel like a failure.
   2. I have failed more than I should have.
   3. As I look back, I see a lot of failures.
   4. I feel that I am a total failure as a person.
3. **Loss of Satisfaction**
   1. I am not particularly dissatisfied.
   2. I am often dissatisfied.
   3. I am usually dissatisfied with most aspects of my life.
   4. I am dissatisfied with every single aspect of my life.
4. **Guilty feelings**
   1. I don’t feel particularly guilty.
   2. I feel guilty over many things I have done or should have done.
   3. I feel quite guilty most of the time.
   4. I feel guilty all of the time.
5. **Punishment feelings**
   1. I don’t feel I am being punished.
   2. I feel I may be punished.
   3. I expect to be punished.
   4. I feel I am being punished.
6. **Self-Dislike/Self-Hate**
   1. I feel the same about myself as ever.
   2. I have lost confidence in myself.
   3. I am disappointed in myself.
   4. I dislike myself.
7. **Self-Criticalness/ Self Accusation**
   1. I don’t criticize or blame myself more than usual.
   2. I am more critical of myself than I used to be.
   3. I criticize myself for all of my faults.
   4. I blame myself for everything bad that happens.
8. **Suicidal Thoughts or Wishes**
   1. I don’t have any thoughts of killing myself.
   2. I have thoughts of killing myself, but I would not carry them out.
   3. I would like to kill myself.
   4. I would kill myself if I had the chance.

**10. Crying**

- 1. I don’t cry anymore than I used to.
  2. I cry more than I used to.
  3. I cry over every little thing.
  4. I feel like crying, but I can’t.

1. **Irritability**
   1. I am no more irritable than usual.
   2. I am more irritable than usual.
   3. I am much more irritable than usual.
   4. I am irritable all the time.
2. **Loss of Interest/ Social Withdrawal**
   1. I have not lost interest in other people.
   2. I am less interested in other people or things than before.
   3. I have lost most of my interest in other people or things.
   4. It’s hard to get interested in anything.
3. **Indecisiveness**
   1. I make decisions about as well as ever.
   2. I find it more difficult to make decisions than usual.
   3. I have lost most of my interest in other people or things.
   4. It’s hard to get interested in anything.
4. **Body Image**
   1. I don’t fell I look any worse than I used to.
   2. I am worried that I am looking worse than I used to.
   3. I feel that I usually look attractive.
   4. I feel that I am very ugly/repulsive looking.
5. **Work Inhibition**
   1. I work as well as usual.
   2. I feel that I do not work as well as I used to.
   3. Working for me is very difficult.
   4. I cannot do any work at all.
6. **Changes in Sleeping Pattern**
   1. I have not experienced any change in my sleeping pattern.
   2. I cannot sleep as well as I used to.
   3. I wake earlier than I used to and have difficulty falling back asleep.
   4. I wake very early and it is impossible to fall back asleep.
7. **Tiredness or Fatigue**
   1. I am no more tired or fatigued than usual.
   2. I get more tired or fatigued more easily than usual.
   3. I am too tired or fatigued to do a lot of the things I used to do.
   4. I am too tired or fatigued to do most of the things I used to do.

**18. Changes in Appetite**

- 1. I have not experienced any change in my appetite.
  2. My appetite is somewhat less usual.
  3. My appetite is much less than before.
  4. I have no appetite.

1. **Weight Loss**
   1. My weight has not changed.
   2. I have lost about 5 kilos.
   3. I have lost about 10 kilos.
   4. I have lost more thank 15 kilos.

**20. Somatic Preoccupation**

- 1. I am not more worried about my health than usual.
  2. I am concerned about my health than I used to be.
  3. I am so concerned about my health it is hard to think about anything else.
  4. The only thing I can think about is my health worries, and nothing else.

1. **Loss of Interest in Sex**
   1. I have not noticed any recent change in my interest in sex.
   2. I am less interested in sex than I used to be.
   3. I am much less interested in sex now.
   4. I have lost interest in sex completely.
