# Supplementary material for: Depressive symptoms and associated factors among persons with physical disabilities in disability care homes of Kathmandu district, Nepal: A mixed method study
Source: PLOS Glob Public Health. 2023 Jan 12;3(1):e0001461. doi: 10.1371/journal.pgph.0001461 (PMC10021957; doi:10.1371/journal.pgph.0001461)
Supplement: S1 Table — (DOCX) [file pgph.0001461.s003.docx]

**S1 Table. Participants Details**

***Characteristics of participants of an in-depth interview.***

| **Participants** | **Age (years)** | **Gender** | **Type of Disability** | **Education Level** | **Employment** | **Assistive Devices** |
| --- | --- | --- | --- | --- | --- | --- |
| P- 1 | 35 | Male | Spinal-cord injury | Secondary level | Self-employed | Wheelchair |
| P- 2 | 25 | Female | Spinal-cord injury | Higher Secondary | Unemployed | Wheelchair |
| P-3 | 63 | Male | Leprosy affected | Informal education | Unemployed | No |
| P-4 | 21 | Male | Leprosy affected | Illiterate | Unemployed | Crutches &  Prosthetic Leg |
| P- 5 | 71 | Male | Leprosy affected | Primary level | Unemployed | No |
| P- 6 | 56 | Male | Leprosy affected | Informal education | Unemployed | No |
| P- 7 | 34 | Female | Stunted | Primary level | Self employed | No |
| P- 8 | 85 | Male | Leprosy affected | Illiterate | Unemployed | No |
