# Supplementary material for: Depressive symptoms and associated factors among persons with physical disabilities in disability care homes of Kathmandu district, Nepal: A mixed method study
Source: PLOS Glob Public Health. 2023 Jan 12;3(1):e0001461. doi: 10.1371/journal.pgph.0001461 (PMC10021957; doi:10.1371/journal.pgph.0001461)
Supplement: Attachment — Submitted filename: Response to Reviewers.docx [file pgph.0001461.s004.docx]

| Dear Editor and Reviewers,  Thank you so much for this wonderful consideration and we highly appreciate your meticulous review and constructive comments and suggestion. We have revised our manuscript incorporating your comments and suggestions. Here we write a rebuttal letter that responds to each point raised by the Editor and Reviewers. Please kindly consider our responses. | |
| --- | --- |
| **Comments** | **Response** |
| **Journal Requirements:** | |
| 1. In the ethics statement in the Methods, you have specified that verbal consent was obtained. Please provide additional details regarding how this consent was documented and witnessed, and state whether this was approved by the IRB. | Written consent was taken for quantitative interview.  And for qualitative data, we have added one sentence in Ethical consideration section of the manuscript.  *Line 177: And verbal consent was recorded while recording the interview for the qualitative study which was approved by IRC.* |
| 2. Please update your online Competing Interests statement. If you have no competing interests to declare, please state: “The authors have declared that no competing interests exist.” | Done during file submission. |
| 3. Please provide a complete Data Availability Statement in the submission form, ensuring you include all necessary access information or a reason for why you are unable to make your data freely accessible. If your research concerns only data provided within your submission, please write “All data are in the manuscript and/or supporting information files.” as your Data Availability Statement. | Done during file submission. |
| 4. Please ensure that you refer to Table 2 in your text as, if accepted, production will need this reference to link the reader to the table. | Yes. We have incorporated as indicated. |
| 5. We notice that your supplementary materials are included in the manuscript file. Please remove them and upload them with the file type 'Supporting Information'. Please ensure that each Supporting Information file has a legend listed in the manuscript after the references list. | Yes. We have incorporated as indicated. |
| **Reviewers' comments:** | |
| **Reviewer#1** | |
| There are grammatical errors in some places, please proofread the document. | Yes. We have incorporated as indicated. We have checked the document with Grammarly software and all authors checked for grammatical errors and tried to solve them. |
| Line 89-95: How is this a “mixed explanatory concurrent study“? The information in the methodology part is confusing. If it is explanatory, will the study be concurrent or sequential? Are you trying to triangulate findings or explain reasons from quantitative studies? | Yes. We have incorporated as indicated.  The updated line.  Line 93: *We used a concurrent triangulation mixed methods design where both quantitative and qualitative data were collected simultaneously and independently.* |
| Line 111: How was Systematic Random Sampling used? | We have explained the process of sampling in details under Study population and sample size heading. The information written is as below:  *Line 114-118:*  *The samples were selected by using a systematic random sampling technique. The list of the all persons with physical disability was obtained from all the care homes of Kathmandu district. The first participant was selected by the lottery method. Then, each study participant was chosen using systematic random sampling where the sampling interval (K) was 2.* |
| Line 112: Why were people more than sample size taken? | Yes. We have incorporated as indicated in line 112. |
| Line 151: Any reason for not taking written consent? | Written and verbal consent was taken from the participants. It was missed in the manuscripts. Now, we have incorporated as indicated. |
| Line 175: Do you mean 0.5 instead of 10.5 %? Check for similar errors? | It was a typo error. We have corrected it. |
| Table 3: Please write what the numbers in parentheses indicate. | Yes. We have incorporated as indicated. |
| Line 208: Why were more male participants chosen for qualitative study? Any specific reason for that? | The reason for most the male (6 interviewees), as male participants were more open and interested in sharing their feelings as compared to women. |
| Line 465: Why were people with multiple disability excluded? Don't you think this may affect the findings? Was this in the eligibility criteria? | Yes. We have incorporated as indicated.  *Line 525-527:*  *Our study did not include persons with multiple disabilities to reduce the non-response rate because of severity and lack of specialized human resources to interview with deaf, deaf-blind or other disabilities.* |
| **Reviewer #2:** | |
| Line 31: This design is not correct. Explanatory design must be sequential and concurrent design must be used to triangulate the findings from qualitative and quantitative findings. Also describe which method was dominant. | Yes. We have incorporated as indicated. Necessary changes has made in abstract and methodology section. |
| Line 33: Does BDI give symptoms only or more? It is used in clinical practice as well so make sure you have used the right tool to measure the depressive symptoms. DASS is used more frequently to measure the symptoms and BDI is used for diagnosis. | Yes. We have incorporated as indicated.  Added necessary citation to address this comment in Line 93-99 in methodology. |
| Line 36: This is a classical concurrent mixed method. It is not a sequential design. | Yes. We have incorporated as indicated. Necessary changes has been made in abstract and method sections. |
| Line 91: Make sure that this is the right tool to investigate depressive symptoms or not as it is also widely used to diagnose depression. | Yes. We have incorporated as indicated.  Added necessary citation to address this comment in Line 125-128. |
| Line 93: No. It was collected simultaneously as described above so this was not based on the sequential mixed method design. Change this accordingly | Yes. We have incorporated as indicated. |
| Line 97-101: Change the title as per this description, do not over generalize. Title should represent what was done and where it was done and to whom it was done. | Yes. We have incorporated as indicated. The title has been revised accordingly. |
| Line 105: Write “minimum sample size”. | Yes. We have incorporated as indicated. |
| Line 106: Why was this proportion used? Provide justification. | The relevant study was not found to incorporate the proportion of previous study. So, with consulting statistician, this proportion was used. |
| Line 120: Change: … scale in Nepal language was … | Yes. We have incorporated as indicated along with necessary citation in line 128. |
| Line 125: Describe clearly who did this and who conducted the interview. How much experience did the interviewer have for conducting this type of interview? | Yes. We have incorporated as indicated.  *Line 131-134: For the qualitative method, the principal investigator himself conducted in-depth interviews who had previous academic and professional experiences in conducting qualitative research and also had experience working for the rights of persons with disabilities.* |
| Line 132-133: Provide reference of both the software. If you have license of the SPSS software mention it too so that it becomes ethical to use it for this research | Yes. We have incorporated as indicated in line 166.  The SPSS was used from the App store of the University. Hence, we currently not able to provide the reference number. We are sorry for this. |
| Line 135-37: Provide reference here. | Yes. We have incorporated as indicated. |
| Line 137-40: How did you decide to take independent variables from bivariate analysis to multivariate analysis. Did you check for confounding using variance inflation factor? If yes then provide how it was used along with the cut-off used for this particular regression model. | Yes. We have incorporated as indicated.  Line 148-156:  Using logistic regression, we performed bivariate and multivariate analysis to observe the association of independent variables, including individual’s socio-demographic characteristics, health behaviors, educational and employment status, with depressive symptoms. Independent variables found statistically significant (p<0.05) in univariate analysis were further utilized in multivariate analysis. Multi-collinearity was checked using the variance inflation factor (VIF>10). The highest VIF was 1.13, which suggested there was no multi-collinearity problem. Statistical testing was done on a 95% significance level, where a p-value of less than 0.05 was considered to be significant. |
| Line 141: Did you use this for taking the variables from bivariate to multivariate model too? If yes, provide justifications. | We used similar approaches for multivariate analysis where we considered p-value <0.05 at 95% significance level. |
| Line 142-46: Which analysis framework was used. How did you ensure the reliability of the coding? Write about the researcher’s reflexivity here. | Yes. We have incorporated as indicated.  Line 165-171:  As part of reflexive thematic analysis, final transcripts were coded by PK in tandem with another investigator (RB) in order to find similar patterns in transcripts. Codes were finalized through peer validation with a third researcher (BS). Each interview was summarized after coding with identified themes and categories of information. . To generate meaning and further develop the themes, we looked over the textual data again. After analyzing quantitative and qualitative data, both results were combined and presented in a joint display[19]. |
| Line 168: Why only? Write Around here as you are not comparing. | Yes. We have incorporated as indicated. |
| Line 193: What are you presenting in the parentheses here in Table 3? | Yes. We have incorporated as indicated. It was Confidence Interval. |
| Line 204: Confounding effect among the factors eligible for the final model was not done so the results presented in Tablie 4 are not valid. | The result of bivariate analysis has been reported in supplementary file name “Bivariate analysis”. Only the result with p value less than 0.05 was included in the final model for Table 4. |
| Line 207: Describe the analysis framework used e.g. conceptual content analysis or relational content analysis. If it was conceptual content analysis then describe which framework was used to analyze the data and arrive at the themes e.g. Braun and Clarke’s six steps for doing thematic analysis. | Yes. We have incorporated as indicated in the line 157-164 in data management and analysis section. |
| Line 212: Describe the process of open coding followed by axial coding and selective coding process used to reach this theme. | Yes. We have incorporated as indicated in qualitative data management and analysis section. |
| Line 371: Organize this as convergent finding, divergent finding and expansive finding. | Yes. We have incorporated as indicated from line 419 in integrated findings. The finding was written in expansion approach. |
| Line 388: Present these results in terms of convergent findings, divergent finding and expansive findings. Read how to mix/triangulate concurrent mixed methods result and do the needful | Yes. We have incorporated as indicated in result section. The finding was written in expansion approach. |
| Line 390: Present these results in terms of convergent findings, divergent finding and expansive findings. Read how to mix/triangulate concurrent mixed methods result and do the needful. | Yes. We have incorporated as indicated. The finding was written in expansion approach. |
| Line 393: Re-write the discussion based on convergent, divergent and expansive findings that will be obtained after you re-analyze your data accordingly. | Yes. We have incorporated as indicated in discussion. The finding was written in expansion approach. |
| Line 465-467: You study is not community based; it is institutions based so highlight it too here. | Yes. We have incorporated as indicated in line 528 as institution based study. |
| Line 468: Re-write the conclusion based on convergent, divergent and expansive findings that will be obtained after you re-analyze your data accordingly. | Yes. We have incorporated as indicated in conclusion. Both quantitative and qualitative data were re-analyzed. |

Thank you,

Corresponding author
