# Supplementary material for: Depressive symptoms and associated factors among persons with physical disabilities in disability care homes of Kathmandu district, Nepal: A mixed method study
Source: PLOS Glob Public Health. 2023 Jan 12;3(1):e0001461. doi: 10.1371/journal.pgph.0001461 (PMC10021957; doi:10.1371/journal.pgph.0001461)
Supplement: Attachment — Submitted filename: Response to Reviewers.docx [file pgph.0001461.s005.docx]

| Dear Editor and Reviewers,  Thank you so much for this wonderful consideration and we highly appreciate your meticulous review and constructive comments and suggestion. We have revised our manuscript incorporating your comments and suggestions. Here we write a rebuttal letter that responds to each point raised by the Editor and Reviewers. Please kindly consider our responses. | |
| --- | --- |
| **Comments** | **Response** |
| **Reviewer #1** | |
| There are still some grammatical and language errors. Please read the document thoroughly | Thanks for your suggestion, we have corrected it. |
| Keywords: Should be in alphabetical order | We have corrected it in Line 49. |
| Table 3: Is this 95 % confidence interval? | Yes.  Line 232 |
| Table 4: Write the meaning of asterisk (*) below the table | We mentioned it in revised version of manuscript in Line 244. |
| **Reviewer#2** | |
| Line 38/39 - This result must be verified after ruling out the multicollinearity among the independent variables. | Yes we verified it. |
| Line 95 - use method instead of methods here as you have used only one type of qualitative method | Yes we corrected it accordingly.  *Line 91: “This is a concurrent design where the quantitative method was dominant and the qualitative method was used to triangulate quantitative results in this study.”* |
| Line 97 - remove the name of the tool used, not required here | Yes we corrected it accordingly. |
| Line 97 - replace investigate with proper action verbs for qualitative and qualitative methods | Yes we corrected it accordingly.  *Line 94: “The study aimed to determine the status of depressive symptoms and explore its associated factors among persons with physical disabilities living in care homes of Kathmandu District.”* |
| Line 98 - replace in with 'living in care homes of' | Yes we corrected it accordingly.  *Line 95:*  *“The study aimed to determine the status of depressive symptoms and explore its associated factors among persons with physical disabilities living in care homes of Kathmandu District.”* |
| Line 103 - It is not clear why other districts are mentioned here, see comments in the manuscript and do accordingly | Yes we corrected it accordingly. The line has been omitted from the manuscript. |
| Line 109 - Sample size is not correct, need re-checking | Yes we rechecked it accordingly in the manuscript as below.  Line 106:  “The required sample size was 162. Based on the formula, n = z^2^pq/e^2^, where *Z* is the standard normal variate (1.96) at a 95 % confidence interval, *p* is prevalence and 0.5 was used for the unknown prevalence[12], *q* is 1-prevalence (0.5), and *e* is the margin of error (0.05), the sample size was 384.” |
| Line 109 - Margin of error is represented by 'e' so use e instead of d in the formula | Yes we corrected it accordingly and replace with “e” in the manuscript. |
| Line 110 - Reference or justification is required for p=0.5 | We did not found the prevalence in similar setting so we decided to use the 50 % for the unknown prevalence % to calculate appropriate sample size for this study as the article which is cited in the manuscript.  Line 108:  “…… *p* is prevalence and 0.5 was used for the unknown prevalence [12]……” |
| Line 111 - Replace d=allowable error with e=margin of error | Yes we corrected it accordingly in Line 106. |
| Line 111 - Value of N in the Finite population correction is missing, you must provide it with proper reference | Yes we corrected it accordingly.  *Line 109:*  *“Further, using finite population correction methods, i.e., n=n/ (1+n/N), where N=256( Total persons with physical disabilities in all the disability care homes of Kathmandu district were 256), we got 154 as a sample size for the study. Moreover, a 5% non-response rate was added to get the final sample size of 162.”* |
| Line 116 - Describe how the list was arranged (see comment in the manuscript for details) | We have incorporated in line 114.  We obtained the list of person with physical disability from all the care homes of Kathmandu district and arranged it alphabetically. |
| Line 117 - Determine how the sampling interval was determined i.e. provide information on n and N used | The sampling interval (K) was determined by dividing the total population by required sample size so in our study N=256 and n=162, so sampling interval was 2.  We have incorporated in Line 116. |
| Line 119 - Eight person is not enough to get saturation for the qualitative study as the qualitative analysis is done independently in the concurrent mixed methods design | We have added rational for the use of 8 samples in the manuscript.  Line 130 –  *Eight persons with disabilities have been selected as Manns et. al. (2019) have conducted the IDIs with eight respondents from not-profit organizations, which is similar setting in this study.*  However, considering the reviewer’s critical feedback, have incorporated it in the limitations section. |
| Line 120 - Why "randomly"? Provide justification | We are sorry for this error. The participants for IDI was selected purposively and incorporated in line120. |
| Line 121 - So, in-depth interviews was the only qualitative method used in this study | Yes, we corrected it accordingly. |
| Line 146 - Add standard deviation after mean | In our descriptive analysis we did not calculate the mean of any variable so we remove it in our manuscript. |
| Line 152 - Replace "uni-variate" with "bi-variate" | Yes we corrected it accordingly. |
| Line 153 - VIF values is not correct for the logistic regression model | Yes we corrected it accordingly. The VIF cutoff value >5 was used for decision on whether variables had collinearity or not.  *Line 155:*  *“*Multi-collinearity of variables was checked using the variance inflation factor (VIF). The highest VIF was 1.54, which suggested there was no multi-collinearity problem [19]. Statistical testing was done on a 95% significance level, where a p-value of less than 0.05 was considered to be significant.” |
| Line 156-57 - The p-value for bi-variate model is different than the multivariate model so change accordingly (see the manuscript for the detail comment) | No, we used same p value for bivariate and multivariable analysis that is 0.05. And those significant variables in bivariate analysis and relevant variables from literature are taken for multivariable analysis. |
| Line 166-67 - Report interceder reliability index here | Yes, we corrected it accordingly. |
| Line 168 - Describe the code learning process, how many were retained and the final interceder agreement | We have incorporated your suggestion and rewrite our data analysis part as follows:  *Line 178 –*  *As part of thematic analysis, final transcripts were coded by PK in tandem with another investigator (RB) with 75% inter-coder agreement in order to find similar patterns in transcripts. Afterwards, a consensus was built to finalize the codes through mutual understanding of coders and peer validation with a third researcher (BS).* |
| Line 172 - No! As per your design, you must present your findings in terms of convergent, divergent and expansive findings | Yes, we corrected it accordingly in the manuscript. |
| Line 241 - No! You can't use the final model before assessing the multicollinearity so do it | Yes, we checked the multicollinearity before multivariable regression and we found there is no concern of multicollinearity |
| Line 247 - Results are biased as multicollinearity was not assessed before using the final model | Thank you so much for your response.  First of all, we did bivariate analysis which result was reported in our supplementary section. We decided to use p value less than 0.05 as significant.  Before deciding for the multivariariable analysis, we checked all the assumption of regression analysis. We checked the multicollinearity using VIF value and found there is no problem of multicollinearity.  After checking multicollinearity, those variable which were significant at bivariate analysis (95% CI, P value less than 0.05) and relevant variables to our study form literature subjected to multivariate analysis. We reported OR with p – value at 95% CI (Table 4). |
| Line 420 - No! You must present your results in three sections here: convergent findings, divergent findings and expansive findings | Yes we corrected it accordingly. We have presented results in convergent and divergent findings and incorporated expansive findings in discussion section. Relevant findings of other literatures are explained within the convergent and divergent findings. |
| Line 438 - You must compare and contrast the findings from convergent, divergent and expansive findings here | Yes we corrected it accordingly |
| Line 533 - You must conclude based on the findings from convergent, divergent and expansive findings. | Yes we corrected it accordingly |

Thank you,

Corresponding author
